# Web-Based and Mobile Delivery of an Episodic Future Thinking Intervention for Overweight and Obese Families: A Feasibility Study

**DOI:** 10.2196/mhealth.4603

**Published:** 2015-12-16

**Authors:** Yan Yan Sze, Tinuke Oluyomi Daniel, Colleen K Kilanowski, R Lorraine Collins, Leonard H Epstein

**Affiliations:** ^1^ Department of Pediatrics Jacobs School of Medicine and Biomedical Sciences University at Buffalo Buffalo, NY United States; ^2^ Department of Community Health and Health Behavior School of Public Health and Health Professions University at Buffalo Buffalo, NY United States

**Keywords:** obesity, ecological momentary intervention, episodic future thinking, Web-based, health behavior

## Abstract

**Background:**

The bias toward immediate gratification is associated with maladaptive eating behaviors and has been cross-sectionally and prospectively related to obesity. Engaging in episodic future thinking, which involves mental self-projection to pre-experience future events, reduces this bias and energy intake in overweight/obese adults and children. To examine how episodic future thinking can be incorporated into clinical interventions, a Web-based system was created to provide training for adults and children in their everyday lives.

**Objective:**

Our study examined the technical feasibility, usability, and acceptability of a Web-based system that is accessible by mobile devices and adapts episodic future thinking for delivery in family-based obesity interventions.

**Methods:**

We recruited 20 parent-child dyads (N=40) from the surrounding community and randomized to episodic future thinking versus a nutritional information thinking control to test the feasibility of a 4-week Web-based intervention. Parents were 44.1 (SD 7.8) years of age with BMI of 34.2 (SD 6.8) kg/m^2^. Children were 11.0 (SD 1.3) years of age with BMI percentile of 96.0 (SD 1.8). Families met weekly with a case manager for 4 weeks and used the system daily. Adherence was collected through the Web-based system, and perceived acceptance of the Web-based system was assessed postintervention. Measurements of body composition and dietary intake were collected at baseline and after the 4 weeks of intervention.

**Results:**

All 20 families completed the intervention and attended all sessions. Results showed parents and children had high adherence to the Web-based system and perceived it to be easy to use, useful, and helpful. No differences between conditions were found in adherence for parents (*P*=.65) or children (*P*=.27). In addition, results suggest that basic nutrition information along with episodic future thinking delivered through our Web-based system may reduce energy intake and weight.

**Conclusions:**

We showed that our Web-based system is an accepted technology and a feasible utility. Furthermore, results provide initial evidence that our system can be incorporated into family-based treatments targeting behaviors related to weight control. These results show promising utility in using our Web-based system in interventions.

## Introduction

The development of obesity is contributed in part by a series of choices that influence positive energy balance. Although it is well publicized that obesity results from energy intake in excess of energy expenditure [1,2], many individuals choose the immediate gratification of overindulging in unhealthy and high-energy-dense foods over the more rational decision of consuming healthier foods for the long-term goal of healthy weight. This choice may reflect the bias toward immediate gratification that has been cross-sectionally and prospectively associated with obesity [3-5]. Moreover, this bias may be compounded for children, who have more difficulty resisting immediate gratification than adults [6], and may be even worse for obese children, who find it more difficult to delay gratification for food rewards [7]. Furthermore, a bias toward immediate gratification predicts diminished success with weight loss in family-based obesity treatment [8].

Skills for reducing the bias toward immediate gratification in adults and children are emerging, and one such skill is episodic future thinking (EFT). EFT, a type of prospective thinking, is mental self-projection into the future to pre-experience events [9]. EFT is thought to reduce impulsive decision making by increasing the value of delayed outcomes [10] and guiding individuals toward choices with long-term benefits [11]. During eating episodes, EFT can reduce the impulsive desire to engage in excess energy intake and reframe time perspective to focus more on healthy weight benefits. Studies show that engaging in EFT reduces the bias toward immediate gratification during decision making [12] and reduces energy intake in tempting food situations in adults [13] and children [14]. However, little is known about EFT’s training effects on eating behaviors outside the context of the laboratory.

A feasible method for delivering EFT was needed to provide daily training in the everyday lives of adults and children. Electronic media offer opportunities to extend current approaches [15,16]. Our Web-based system, the Mobile Audio Manager and Response Tracker (MAMRT), was developed to provide daily training for adults and children during real-world eating episodes in an ecological momentary intervention (EMI). Based on the Technology Acceptance Model [17,18], our Web-based system was designed to be responsive and mobile to increase ease of use, a component necessary for a technology to be accepted.

EMI has become more widely accepted as the use of technology has become ubiquitous in the lives of adults and children [15]. In contrast to controlled laboratory settings, EMI provides treatments to people in their natural settings during their everyday lives, which enhances validity [15]. Furthermore, research shows that interventions incorporating technology can elicit behavioral changes and be effective for changing a variety of health behaviors [19-21]. Previous research has also supported the use of Web-based dietary intervention [22,23].

We incorporated EFT into a Web-based EMI involving 20 parent-child dyads interested in changing their eating behaviors and losing weight. All families were provided 4 weeks of nutrition education and randomized to EFT or a control nutrition information thinking (NIT) condition. NIT was only provided with nutritional education, but no EFT training. Families in both conditions were asked to use the system in the same fashion. We expected no between-group differences in the feasibility, usability, and acceptability of MAMRT. However, because EFT could be helpful in regulating eating, we also explored EFT’s effects in weight change and eating habits compared to the control condition.

## Methods

### Participants and Procedures

Families were recruited from the surrounding community through an existing database and advertisements via flyers and postcards. Parents of interested families were screened by telephone to assess initial eligibility. Families were initially eligible if the parent was overweight and the child was overweight and between the ages of 8 and 12 years. In addition, both had to have access to a mobile electronic device (eg, mobile phone, tablet, laptop) and be interested in weight loss, willing to participate in a 4-week intervention, and not currently involved in other weight loss programs. Initially eligible families were invited to an orientation and a formal screening appointment where both parent and child received a detailed presentation on study procedures, signed consent forms, completed measures, participated in individual interviews, and verified that their electronic devices were compatible with our study purposes. Parents and children were required to have regular access to a mobile electronic device (specifically around eating episodes); however, it was not required that parents and children had their own individual devices. Families were excluded from participation if either the parent and child or both were not considered overweight (body mass index [BMI] less than 25 kg/m^2^ and BMI percentile less than 85), were not interested in changing their eating habits through the use of this technology, were not comfortable using or did not have access to a mobile electronic device with Wi-Fi and/or reliable Internet access, were unable to read at the 3rd grade reading level, or could not follow study protocol (ie, attend all sessions, access electronic device daily, and complete all assessments).

**Figure 1:**
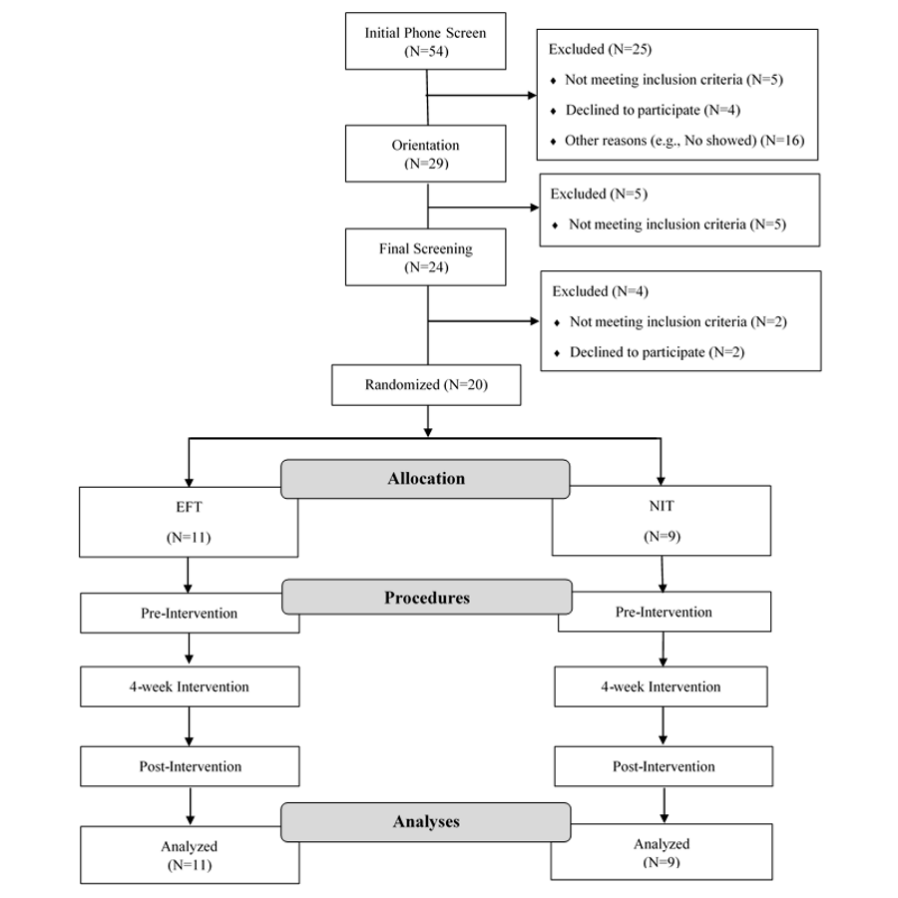
Consort participant recruitment and retention diagram.

A total of 54 parents were initially screened by telephone, and 25 families were excluded. Of the 25 families excluded, 5 families did not meet inclusionary criteria, 4 families declined to participate, and 16 families did not attend orientation. Of the 29 families that attended orientation, 5 families did not meet weight inclusionary criteria and did not continue onto final screening. After final screening, 4 families were excluded. Of the 4 families excluded, 2 families declined to participate and 2 families did not meet final screening inclusionary criteria (Figure 1).

A total of 40 eligible and interested participants (20 overweight/obese parent-child dyads) were invited to participate in the 4-week intervention and were randomly assigned to either the EFT condition (n=11) or the NIT control condition (n=9). Families were randomly assigned to conditions in blocks of 4 families stratified by child sex. Stratification was accomplished based on a sequence set up by the project statistician. The project statistician was not involved in the data collection, and study personnel had no input in the randomization sequence. Participants were not told which group was the experimental group.

Due to the behavioral nature of the manipulations, it was not possible to blind study participants or study personnel to randomization assignment. Outcome assessors who collected weight, dietary intake, and system ratings were not blinded to the intervention; however, outcomes collected electronically such as adherence, dietary restraint, demographics, adherence, and device usage did not require blinding. Parents and children in both conditions completed the same measures, were given the same dietary information, and were instructed to use MAMRT in the same fashion. The difference between the two conditions was families in the NIT control condition received only nutritional information while families in the EFT condition received both nutritional information and EFT training.

Families in both conditions completed 3 days of 24-hour dietary recalls together (parent and child) over the phone before and after the 4-week intervention. Families attended a 90-minute preintervention appointment, 4 weekly 60- to 90-minute intervention sessions, and a 90-minute postintervention appointment. All procedures were conducted in accordance with guidelines for the ethical conduct of human research outlined by the National Institutes of Health and with the approval of the University at Buffalo Social and Behavioral Sciences Institutional Review Board.

At preintervention, parents and children were taught the Traffic Light diet [24]. The Traffic Light diet is an eating plan that categorizes food based on nutrient composition into green, yellow, or red foods. Green foods are low in calories, fat, and sugar and are also rich in nutrients. Yellow foods are higher in calories than green foods but are still a good source of nutrients and include the dietary staples needed for a balanced diet. Red foods are high in calories, fat, and/or sugar and are not a good source of nutrients. All families were taught to eat fewer calories than they normally eat, maintain nutrient balance by eating the recommended servings based on the Dietary Guidelines for Americans [25] issued jointly by the US Department of Agriculture and the US Department of Health and Human Services, limit their consumption of red foods, and increase their consumption of green foods. Parents and children in both conditions were given a workbook to record their daily dietary intake before their first intervention session. All materials were presented at a 3rd grade reading level for adequate comprehension.

**Figure 2:**
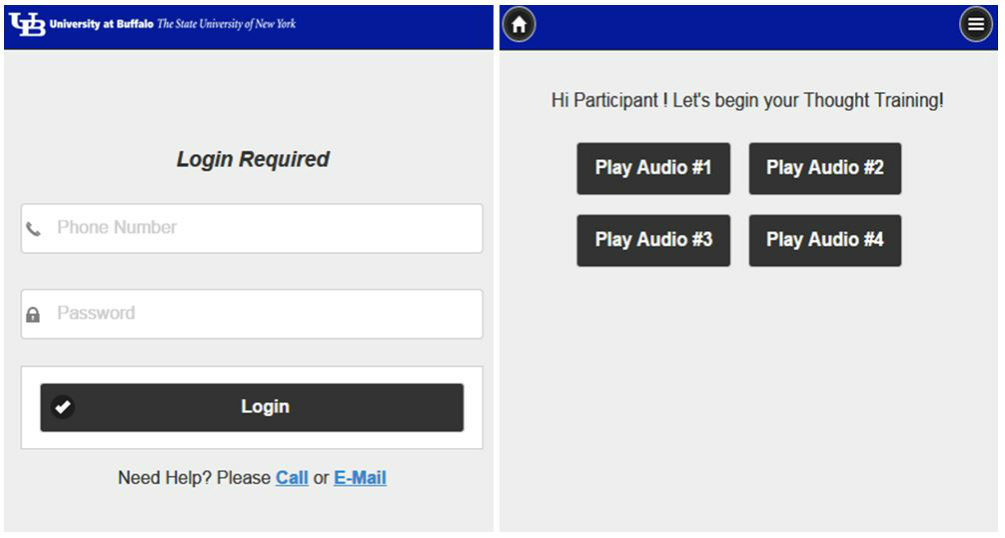
Participant log-in and default landing page (audio page).

At the first intervention session, all families received an introduction on how to use the study website. Parents and children learned how to use their own devices to access the website. The website was developed to be personally tailored and interactive. The opening page was the participant’s log-in page. Each participant (parent and child) was given an individual account that required a unique log-in to access the study website. Upon logging in, participants were directed to an audio page which displayed their cues and their first name (Figure 2).

Cues were participant-generated personal audio recordings created in their own words and recorded in their own voice. During their intervention sessions, families in both conditions generated nutritional cues based on behavioral and dietary strategies known to positively influence weight control such as portion control, energy density, stimulus control, eating out, food shopping strategies, and social support. Participants were told the audio recordings were to be used as cues to help them think about healthy eating behaviors. All families were given the same behavioral and nutritional dietary strategies.

In addition to the behavioral and dietary strategies, parents and children in the EFT condition generated audio recordings of positive events they were looking forward to and could vividly imagine. EFT participants generated cues similar to those used in previous EFT study procedures [12-14]. To help participants think about autobiographical details of their events, they rated the valence, salience, arousal, frequency, and vividness of each event on scales of 1-5 (1 for very low and 5 for very high). Because vividness of episodic imagery in EFT predicts the amount of reduction in bias toward immediate gratification [26], only specific events with the highest scores were recorded. Only EFT participants generated recordings of positive future events.

In both conditions, parents and children generated cues together with a case manager; however, participants generated their own individual recordings. Audio recordings were generated using standard laptop microphones and Audacity, a free, open source, cross-platform software program for recording and editing sounds. Families in both conditions generated 4 audio recordings per session. These recordings generally lasted no more than 60 seconds.

In addition to generating cues at their intervention sessions, individual families in both conditions met together (parent and child) with a case manager to obtain weight measurements and discuss eating habits and use of the Web-based system. Case managers elicited participants’ understanding of the system and emphasized the importance of listening and thinking about their cues during eating episodes. Families in both conditions were directed to use their cues at least twice a day, at any point where decisions were made regarding food (eg, foods that they may eat, are planning to eat, or are actually eating).

To remind participants to use their cues, they received prompts at 6:00 AM and 3:00 PM daily. These prompt schedules were set at those times so that participants would receive them before their first and last meal consumptions. Prompts were delivered automatically through the system and were sent directly to the participant’s electronic device in a text message or email (based on preference). Each reminder included the study website’s URL and a brief message to complete prompt exercises by the end of the day.

Prompt exercises included participants listening to their cues and completing a short survey about their cues. After listening to their audio, participants had the option to listen to more recordings or be directed to a short survey. When participants decided to continue to the survey, they would answer 5 questions that were short responses or multiple choice. There were 2 fixed questions, “What meal did you use your audio for?” and “How are you keeping in mind the cue during the day?” The other 3 questions displayed would change each time the participant accessed the survey to keep the questions novel and nonrepetitive. Examples of other questions were “Thinking about your cue, what’s most interesting about it?” and “How can the cue help you make healthy food choices?” and “How much do you think about your audio cue throughout the day?” These questions were asked to help participants think about their cues and keep their cues in mind during eating episodes.

For example, a participant in the EFT condition may have used an upcoming party as the basis for one EFT cue. In the cue, the participant described the party in detail to help vividly imagine pre-experiencing the event. Before dinner, the participant would listen to that recording, answer questions about the event, and imagine being at that event. The participant would imagine looking good in a specific outfit at the event and how reducing the intake of high-energy-dense foods would help achieve this.

After the 4-week intervention, families attended a 90-minute postintervention appointment where feasibility of MAMRT was evaluated. Parents and children completed evaluations separately. Families were compensated US $30 for completing all 6 dietary recalls, US $96 for complete adherence to study protocol (US $0.50 for each completed exercise with the system plus an opportunity to earn a US $5.00 bonus per week if all exercises were completed by their next appointment), and US $24 for completing measures at their postintervention appointment.

### The Mobile Audio Manager and Response Tracker

**Figure 3:**
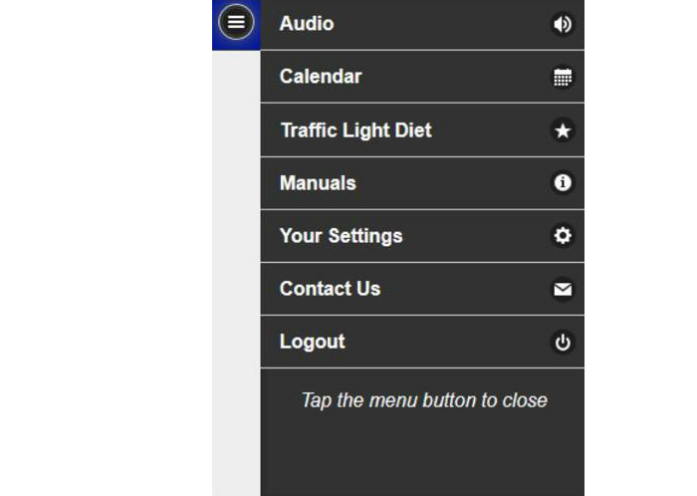
Participant menu page.

The participant interface and investigator page were created using JavaScript, HTML, and CSS. In addition to the audio page, participants could access other features on the study website through the menu tabs (Figure 3). Participants could view their appointment dates and times and the prompt delivery schedule via the Calendar tab, receive instructions and tips on how to use the system through the Manual tab, and contact the experimenters directly with problems through the Contact Us tab. These features allowed study information to be easily accessible for all participants in both conditions.

**Figure 4:**
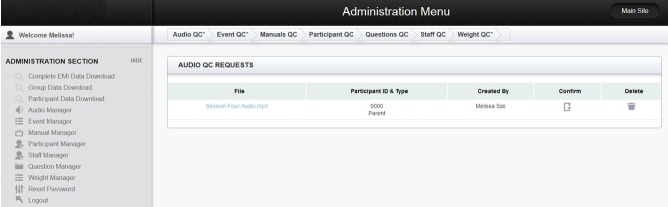
Administrator menu page.

An administrator page linked to the participant’s study website was created as a platform for investigators. To restrict access to investigators only, log-in was required. The administrator page consisted of multiple managers for the various components of the EMI (Figure 4). Investigators added participants and assigned personalized identification numbers, passcodes, condition, and access through the Participant Manager. Through the Audio Manager, investigators could upload specific audio recordings from each session, determine the number of audio files accessible to participants, and view all the audio files that were uploaded. The Event Manager allowed investigators to set up the prompt delivery schedules and participant appointment dates and times that could also be viewed through the participant user interface. In addition, investigators could upload manuals for participants through the Manual Manager, restrict or grant investigator access through the Experimenter Manager, add or remove survey questions in the Question Manager, and enter and view data through the Data Manager. All information added through the administration page would first go to the Quality Check page to ensure that all manually entered information was correct. The administrator page was accessible from a desktop or mobile device allowing investigators to easily monitor the system remotely at any time.

**Figure 5:**
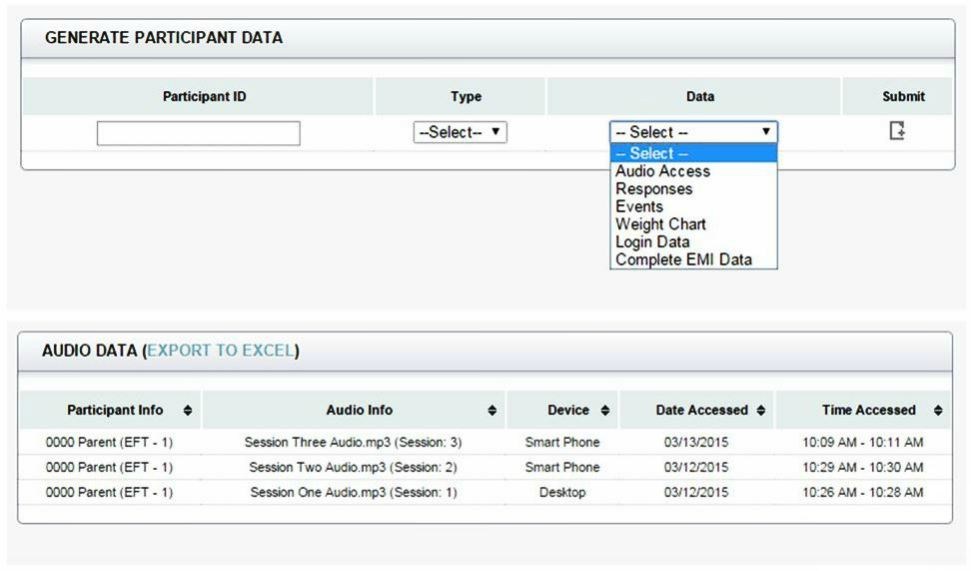
Administrator data manager page.

Data collected from the website were stored and managed in a secure MySQL database only accessible to members of the lab. MySQL is a free, open source, relational database management system based on structured query language. MySQL was used because it is free, stores large amounts of information, and organizes data in a way that can be accessed quickly and easily. The study website collected participant log-in dates and times, device used (eg, mobile phone, tablet, desktop), names of audio file accessed as well as the duration of time it was accessed, and survey responses and the time the responses were submitted. The user interface displayed data in a simple, user-friendly manner. Data could also be downloaded in standard Microsoft Excel format. The system allowed for flexible retrieval of data to address specific questions based on the researcher’s needs (Figure 5).

The system was designed to streamline data collection and analyses for investigators while also being personally tailored and interactive for participants. The system was responsive and compatible with a variety of electronic devices (eg, desktops, laptops, tablets, mobile phones) and operating systems (Windows and OS X).

### Measures

#### Demographics

Race/ethnicity, income, and educational level were obtained using a standardized questionnaire adapted from the MacArthur Research Network on Socioeconomic Status and Health [27] at screening.

#### Anthropometrics

Weight was measured to the nearest 0.2 lb using a digital scale (Tanita BWB-800P), and height was measured in centimeters to the nearest millimeter using a Digi-Kit digital stadiometer. Measurements were taken at each visit. BMI (kg/m^2^), BMI percentile, and percent overweight were established by comparing BMI population standards [28,29]. Overweight/obese children had a BMI at or above the 85th percentile, and parents had a BMI at or above 25.

#### Dietary Restraint

The Dutch Eating Behavior Questionnaire (DEBQ) was completed during screening and used to assess eating behaviors in adults [30]; an adapted version was used for children [31]. The DEBQ is a validated measure to detect 3 different psychologically based eating behaviors: (1) restrained, (2) emotional, and (3) external eating [30,32].

#### Adherence to Using the Mobile Audio Manager and Response Tracker System

Participants were instructed to use the system at two separate periods during the day for 4 consecutive weeks, for a total of 14 responses per week and 56 responses across the 4 weeks. Adherence was collected through the MAMRT system and calculated as the percentage of completed responses daily each week for 4 weeks.

#### Frequency of Device Type Used

The frequency of which device (eg, desktop, tablet, mobile phone) participants used to access the system was collected through the MAMRT system and calculated as the percentage of overall use.

#### Ratings of the Mobile Audio Manager and Response Tracker System

Perceived usefulness, ease of use, and helpfulness were obtained using a self-report survey at postintervention. Parents and children provided ratings on 5-point Likert scales on the usefulness (1 for not at all and 5 extremely) of each component of the study website: (1) listening to nutritional audio cues, (2) completing the survey responses, (3) timing of the prompt reminders, (4) receiving the prompts twice daily, and (5) having 1 Web link to access the audio cue and survey questions. Parents and children provided ratings on how frequently (1 for never and 5 for always) they (1) listened to their audio cues before eating episodes, (2) thought about their audio cue during eating, and (3) used their audio cue in difficult food situations. Rated frequency was used as an index to calculate perceived ease of use. Parents and children provided ratings on how helpful the program was in making healthy changes to their eating habits (1 for not at all and 5 extremely). The program consisted of using the system daily during any eating episode (eg, breakfast, lunch, dinner, snack) and tempting food situations.

#### Dietary Measures

Usual energy intake and servings of green and red foods were accessed through phone-based 24-hour dietary recalls (1 weekend day and 2 weekdays) for parents and children preintervention and postintervention. Parents and children completed their recalls together. Children completed their recalls first with the assistance of their parents, and parents completed their recalls after. The experimenter guided the participants through the dietary recall process using a multipass interview style [33]. First, participants made a quick list of all foods and beverages consumed throughout that day and the time of consumption. For the second pass, the experimenter returned to the beginning of the list and asked for portion sizes, brand names, and the type of foods and beverages that were consumed (regular/diet, white/wheat, etc). The final pass was to prompt the participant to recall other foods they may have forgotten, such as foods that were eaten in small amounts. Participants were asked to have measuring cups, measuring spoons, and a ruler in front of them during the interview. This methodology has been validated in other studies [33,34]. The total number of calories consumed was calculated for the recall based on manufacturer labels and analyzed through Nutritionist Pro software (Axxya Systems LLC) [35]. The reliability of coding red and green foods was determined by 2 independent analysts; the reliability between observers was 94.6% agreement.

### Analytical Plan

Separate one-way analyses of variance (ANOVAs) were conducted to determine group differences in participant characteristics for parent and child. Analyses of device frequency, ease of use, usefulness, helpfulness, and adherence to the system were assessed using a two-way ANCOVA, with group as the between variable and parent education as the covariate. Changes in weight and eating behavior were assessed using three-way ANCOVA, with group as a between variable, pre to post as within variables, and parent education as the covariate. In both analyses, contrasts were used to assess changes by group for parent or child. Data analyses were completed using SYSTAT version 11 (Systat Software).

## Results

All 20 parent-child dyads (N=40) completed the 4 weeks of intervention and attended all sessions (including pre- and postintervention); all were included in the analyses. Parents were 44.1 (SD 7.8) years of age and 90% (18/20) female with a 34.2 (SD 6.8) kg/m^2^ BMI, 15.7 (SD 2.3) years of education, and US $89,705.88 (SD US $58,429.19) household income. Children were 11.0 (SD 1.3) years of age and 45% (9/20) female with a 96.0 (SD 1.8) BMI percentile. There were no significant differences in participant baseline characteristics between the two conditions as shown in Table 1.

Analyses showed a significant overall difference in helpfulness for parents (*F*_1,17_=5.12, *P*=.04), as parents in EFT reported higher helpfulness. There were no significant differences between conditions for parents’ and children’s mean ratings of ease of use (*F*_1,17_=1.45, *P*=.25; *F*_1,17_=1.03, *P*=.32), usefulness (*F*_1,17_=0.21, *P*=.66; *F*_1,17_=0.24, *P*=.63), and mean ratings of children’s helpfulness (*F*_1,17_=1.39, *P*=.25). No significant differences in any of the other variables to assess utility of the system were observed for parents or children as shown in Table 2.

Exploratory analyses for energy intake, red and green food intake, BMI, and percent overweight by parent-child and group are shown in Table 3. Results showed significantly greater reductions in BMI (*F*_1,17_=8.83, *P*=.01) and percent overweight (*F*_1,17_=8.99, *P*=.01) for parents in EFT versus control conditions. In addition, there were trends in larger reductions in energy intake for EFT versus control for both parents (*F*_1,17_=3.05, *P*=.10) and children (*F*_1,17_=3.93, *P*=.06). No other variables showed trends toward significance between group differences.

**Table 1:** Participant baseline characteristics.

		Child			Parent		
		EFT (n=11)	NIT (n=9)	*P* value	EFT (n=11)	NIT (n=9)	*P* value
Age (years), mean (SD)		11.1 (1.4)	11.0 (1.3)	.91	45.0 (7.1)	43.0 (8.8)	.57
Weight (lbs), mean (SD)		139.5 (28.7)	140.6 (38.8)	.94	203.6 (39.7)	205.3 (47.0)	.93
BMI (kg/m^2^), mean (SD)		26.6 (3.7)	27.3 (4.5)	.70	33.5 (6.6)	35.0 (7.4)	.62
BMI percentile, mean (SD)		95.7 (2.1)	96.4 (1.4)	.37	93.2 (4.5)	93.6 (5.8)	.86
Percent overweight, mean (SD)		52.3 (18.7)	57.3 (22.0)	.59	52.5 (30.5)	61.4 (34.0)	.55
Usual calorie intake, mean (SD)^a^		2308.6 (719.5)	1923.7 (463.6)	.18	2153.3 (455.6)	1878.8 (382.4)	.17
Red food servings, mean (SD)^a,b^		22.3 (8.0)	21.7 (5.1)	.85	24.2 (8.8)	23.9 (4.0)	.93
Green food servings, mean (SD)^a,b^		0.7 (1.2)	0.7 (0.9)	.90	0.6 (0.8)	0.2 (0.4)	.30
**DEBQ, mean (SD)** ^c^							
	Restraint	1.8 (0.5)	1.7 (0.5)	.40	3.0 (0.6)	2.8 (0.6)	.59
	Emotional	1.3 (0.5)	1.1 (0.1)	.19	3.1 (0.5)	3.0 (1.0)	.93
	External	2.1 (0.4)	1.8 (0.5)	.10	3.7 (0.4)	3.4 (0.4)	.10
**Sex**				.37			.20
	Male	5	6		2	0	
	Female	6	3		9	9	
**Race/ethnicity**				.78			.89
	Non-minority, non-Hispanic	8	6		10	8	
	Other race/ethnicity	3	3		1	1	
**Education**							.09
	Some high school				0	1	
	High school				0	1	
	Some college or vocational training				1	0	
	Completed 2-year college degree				2	2	
	Completed 4-year college degree				4	4	
	Compete graduate degree				6	1	
**Income** ^d^							.17
	US $49,999 or less				1	2	
	US $50,000-89,999				4	3	
	US $90,000-139,999				2	3	
	US $140,000 or more				2	0	

^a^Assessed by 24-hour dietary recalls.

^b^Servings are based on the USDA Dietary Guidelines for Americans.

^c^Dutch Eating Behavior Questionnaire.

^d^A total of 3 participants chose not to answer this item.

**Table 2:** Adherence, device frequency, and ratings of the MAMRT system.

		Child			Parent		
		EFT (n=11)	NIT (n=9)	*P* value	EFT (n=11)	NIT (n=9)	*P* value
**Adherence, n % (SD)**							
	4-week average response	78.7 (15.1)	81.0 (15.5)	.65	87.0 (13.3)	92.9 (8.5)	.27
**Device frequency, n % (SD)** ^a^							
	Desktop	27.0 (30.9)	16.1 (33.0)	.34	34.2 (38.9)	21.3 (33.7)	.33
	Tablet	21.4 (28.7)	31.2 (37.0)	.24	2.7 (5.9)	16.5 (31.3)	.15
	Mobile phone	51.7 (39.2)	52.8 (44.4)	.87	63.1 (38.5)	62.2 (45.1)	.92
**Ease of use, mean (SD)** ^b^							
	Listening to audio cue before eating	3.5 (0.7)	3.6 (1.5)	.97	3.4 (0.7)	3.9 (0.8)	.41
	Thinking about events from audio cue during eating episodes	3.6 (0.9)	3.1 (1.6)	.37	3.8 (1.2)	3.6 (0.9)	.26
	Using audio cue in tempting situations	3.6 (1.0)	2.8 (1.5)	.18	3.5 (1.1)	2.7 (0.9)	.08
**Usefulness, mean (SD)** ^b^							
	Nutrition audio cue	3.4 (0.9)	3.4 (1.5)	.79	4.0 (1.0)	4.0 (1.0)	.62
	Completing survey responses after listening to the audio cue	3.7 (1.2)	3.6 (1.6)	.67	2.5 (1.2)	3.1 (1.5)	.76
	Timing of the scheduled prompts and reminder messages	2.8 (1.1)	3.0 (1.7)	.87	3.7 (0.9)	3.3 (0.7)	.32
	Receiving prompts twice daily	3.8 (0.9)	3.2 (1.8)	.39	4.1 (1.1)	3.9 (0.6)	.80
	One web link to access audio cue and survey questions	3.8 (1.3)	3.3 (1.2)	.54	4.0 (1.4)	4.0 (1.3)	.75
**Helpfulness, mean (SD)** ^b^							
	Changing eating habits	4.2 (0.9)	3.9 (1.1)	.25	4.3 (0.8)	3.9 (0.9)	.04

^a^Overall frequency of which device was used to access the system.

^b^Ratings of the system are on a 5-point Likert scale (values closer to 1=not at all; 5=extremely).

**Table 3:** Changes in dietary intake and body composition.

	Children			Parents		
	EFT (n=11)	NIT (n=9)	*P* value	EFT (n=11)	NIT (n=9)	*P* value
Usual calorie intake, mean (SD)^a^	−839.6 (650.7)	−443.2 (423.4)	.06	−791.2 (348.3)	−482.4 (237.3)	.10
Red food servings, mean (SD)^a,b^	−8.6 (7.2)	−6.8 (3.3)	.31	−7.5 (6.0)	−6.4 (3.0)	.88
Green food servings, mean (SD)^a,b^	0.8 (1.3)	0.8 (1.2)	.33	0.5 (0.8)	0.1 (0.3)	.19
BMI (kg/m^2^), mean (SD)	−0.6 (0.5)	−0.4 (0.6)	.61	−1.0 (0.5)	−0.2 (0.5)	.01
Percent overweight, mean (SD)	−3.8 (3.1)	−2.7 (3.5)	.67	−4.6 (2.0)	−1.1 (2.2)	.01

^a^Assessed by 24-hour dietary recalls.

^b^Servings are based on the USDA Dietary Guidelines for Americans.

## Discussion

### Principal Findings

This study examined the feasibility, usability, and acceptability of our Web-based system, MAMRT, for dietary interventions. According to the Technology Acceptance Model, perceived usefulness and ease of use are determinants of acceptance and use of a certain technology [17]. Parents’ and children’s high ratings of ease of use and usefulness in both conditions suggest that the system was an accepted technology. Moreover, high ratings of helpfulness and high adherence suggest that our Web-based system is a feasible utility for interventions in both adults and children. No differences found between groups in ratings of ease of use and usefulness of the system and participant adherence suggest that our system is highly usable and may be adapted to serve a variety of EMI purposes (not limited to EFT or nutritional guidance).

To our knowledge, this is the first technology with a responsive design for users and investigators that collects detailed time-stamped usage of audio files including the duration accessed and the device type used (eg, mobile phone, tablet, desktop) to access those files, includes an automated messaging delivery service, and organizes and displays data based on investigator needs. These specifications were necessary in determining participant usability and acceptability and streamlining data collection for the investigators.

While the MAMRT system had several components (prompts, audio files, and database), the cost of execution was relatively low. The supporting software used (MySQL and Audacity) is free, and the creation of additional users (participants/investigators) required no cost. Furthermore, the portability and compatibility with various platforms and ability to modify survey questions suggest translation to other research topics and settings (eg, multisite).

Although our study was not powered to detect meaningful difference in changes in weight and energy intake, results favored the EFT participants in reduction of parent weight and trends toward significant differences in energy intake. These results support research demonstrating the effect of EFT in reducing the bias toward immediate gratification [10,12-14,26] that can be related to obesogenic behaviors [36,37]. While this feasibility study was only implemented for 4 weeks, these exploratory analyses supports future research in investigating MAMRT’s use in interventions for changing unhealthy eating behaviors.

### Strengths and Limitations

There are potential limitations in this pilot feasibility study. We excluded families without access to mobile electronic devices, which could potentially bias our sample. For example, adherence rates to the system could decrease for families with lower socioeconomic status (SES). Although our sample size was small, predominately white, and well-educated, electronic technologies have become more available, affordable, and widely adopted across all SES and age groups in the United States [38,39]. Furthermore, a strength to MAMRT is the flexibility for participants. Whereas some EMIs require mobile phones, short message service, and/or the use of certain operating systems, our system is not limited to any specific device, permitting participants to use the system at no additional cost. The flexibility of the system allows it to be available for all user demographics and diverse groups of participants. Moreover, delivery of the prompts (email or text) did not influence responses (eg, adherence, speed).

The 4-week use of the MAMRT is short, and its use was restricted by environmental limitations. Unlike parents, who generally had access to their electronic devices throughout the day, children were limited due to school and other obligations. Considering that some children consumed 2 of the 3 main meals during school, this restriction limited children’s opportunity to use the system during those eating episodes. We also did not exclude individuals who did not have their own electronic devices. In the future, it would be interesting to see if requiring both parents and children to have their own mobile devices would increase EFT exposure and produce greater behavioral changes because results showed both parents and children used mobile phones more frequently than other devices to access the system.

Regardless of the small sample size and short duration of the intervention, we did find significant changes in parental weight and trends in energy intake favoring EFT for parents and children from pre- to postintervention. It is interesting that parents but not children reported the EFT system to be more helpful than control. Understanding the basis for these generational differences in perception of helpfulness may be important to design more powerful EFT interventions as complements to obesity treatment in the future.

It is plausible that children in the EFT condition were unable to master all the new dietary information introduced in that short amount of time in conjunction with learning EFT. Perhaps EFT children were overwhelmed by having to simultaneously learn nutritional information and the use of EFT. In a longer program, it may be beneficial to phase in EFT after the basic aspects of family-based weight control treatment have been mastered. Further research is needed to investigate if increased exposure to EFT and MAMRT would produce greater behavioral changes.

Other studies have demonstrated the feasibility of using technology in weight control [40,41] and even suggested that EMI enhances standard treatments [42,43]. As family-based treatments last for considerably longer than 4 weeks, it is important to investigate if increased exposure to MAMRT and greater exposure to elements of traditional family-based treatments (eg, dietary information, exercise program, positive parenting) would produce greater and more sustained weight loss and changes in eating behavior. One advantage of the use of technology to implement EFT is that EMI can reduce the burden that may accompany traditional in-person treatment meetings. These advantages include daily support, reduced expenses (eg, travel) and participation in interventions, and personally tailored programs for participants. Tailoring programs may increase effectiveness of health behavioral interventions [44,45]. Moreover, our system could be a suitable replacement for dietary recording, which can be time consuming and challenging for participants. It is possible that the use of the MAMRT system may reduce the need for as many in-person meetings as traditional family-based treatment, making the treatment more accessible and powerful.

### Conclusion

This study examined the feasibility of a Web-based intervention for children and adults that adapts EFT for delivery of family-based obesity treatment. These results demonstrated that both adults and children found MAMRT usable and acceptable; adherence to the system demonstrated its feasibility, consistent with the Technology Acceptance Model [17,18]. Future research is needed to determine its utility in behavioral change. These findings have implications for family-based interventions that attempt to improve eating behaviors in adults and children simultaneously, in the real world and in real time.

## Abbreviations

ANOVA: analysis of variance
BMI: body mass index
DEBQ: Dutch Eating Behavior Questionnaire
EMI: ecological momentary intervention
EFT: episodic future thinking
MAMRT: Mobile Audio Manager and Response Tracker
NIT: nutritional information thinking
SES: socioeconomic status
